# Synthesis of Monodispersed Ag-Doped Bioactive Glass Nanoparticles via Surface Modification

**DOI:** 10.3390/ma9040225

**Published:** 2016-03-24

**Authors:** Dominika Kozon, Kai Zheng, Elena Boccardi, Yufang Liu, Liliana Liverani, Aldo R. Boccaccini

**Affiliations:** 1 Institute of Biomaterials, Department of Material Science and Engineering, University of Erlangen-Nuremberg, 91058 Erlangen, Germany; kozon.dominika@gmail.com (D.K.); elena.boccardi@fau.de (E.B.); liliana.liverani@fau.de (L.L.); aldo.boccaccini@ww.uni-erlangen.de (A.R.B.); 2 Faculty of Chemistry, Warsaw University of Technology, 00-664 Warsaw, Poland; 3 Food Chemistry Unit, Department of Chemistry and Pharmacy, University of Erlangen-Nuremberg, Schuhstr. 19, 91052 Erlangen, Germany; yufang.liu@fau.de

**Keywords:** bioactive glass nanoparticle, bioactivity, silver, surface modification, antibacterial activity

## Abstract

Monodispersed spherical Ag-doped bioactive glass nanoparticles (Ag-BGNs) were synthesized by a modified Stöber method combined with surface modification. The surface modification was carried out at 25, 60, and 80 °C, respectively, to investigate the influence of processing temperature on particle properties. Energy-dispersive X-ray spectroscopy (EDS) results indicated that higher temperatures facilitate the incorporation of Ag. Hydroxyapatite (HA) formation on Ag-BGNs was detected upon immersion of the particles in simulated body fluid for 7 days, which indicated that Ag-BGNs maintained high bioactivity after surface modification. The conducted antibacterial assay confirmed that Ag-BGNs had an antibacterial effect on *E. coli*. The above results thereby suggest that surface modification is an effective way to incorporate Ag into BGNs and that the modified BGNs can remain monodispersed as well as exhibit bioactivity and antibacterial capability for biomedical applications.

## 1. Introduction

Bioactive glasses (BGs) are promising biomaterials for bone regeneration due to their osteoconductivity and osteoinductivity. The first developed BG, well-known as 45S5 Bioglass^®^, is based on the SiO_2_-P_2_O_5_-CaO-Na_2_O composition [1]. Further developments in BG compositions, such as the introduction of therapeutic ions (e.g., Ag^+^, Cu^2+^ or Sr^2+^), can bring new advantages and novel applications to conventional BGs, including, soft tissue regeneration, wound healing, and antibacterial applications [2,3].

Bioactive glass nanoparticles (BGNs) have advantages over bulk BGs for biomedical applications due to their larger surface area, higher bioactivity, as well as controllable and homogenous size [4]. BGNs are usually synthesized by sol-gel-based methods [4]. However, preparation of monodispersed BGNs is still challenging, as the addition of salt precursors may interfere with the nucleation and growth of primary silica particles, consequently leading to irregular and aggregated particles [5]. To synthesize monodispersed BGNs, the type and amount salt precursors (e.g., calcium salts) must be carefully controlled [6,7].

Infection is one of the challenges during implantation, which can cause implant failure or tissue necrosis [8,9]. Incorporating antiseptic ions into the composition of materials is a potential alternative to treat or prevent infections, otherwise carried out by the administration of antibiotics. It is well known that silver (Ag) has broad-spectrum antibacterial activity; therefore, Ag^+^ ion release is a popular approach investigated in the context of antibacterial biomaterials [10,11,12].

Silver-doped BGNs (Ag-BGNs) can exhibit the bioactive characteristics of BGNs as well as showing antibacterial capacity and are therefore investigated for various applications. However, most Ag-BGNs introduced in previous reports appeared to be highly aggregated [13,14,15], which may limit their application as building blocks for development of nanocomposites. Surface modification is a convenient approach to introduce Ag ions into glasses [16]. Monodispersed Ag-doped silica particles have been synthesized by such surface modification methods using silver nitrates, and they have shown considerable antibacterial effects [17].

Based on the know-how available in the literature, we hypothesized that BGNs could adsorb Ag^+^ ions by a surface modification routine and still be monodispersed. Sol-gel-derived BGNs usually have high concentration of silanol groups and negative net charges at the surface [5], which may be beneficial to the adsorption of positively charged Ag^+^ ions. The aim of this research was to prepare monodispersed Ag-BGNs exhibiting bioactivity and antibacterial properties by surface modification at relatively low temperatures. Monodispersed BGNs were synthesized by a modified Stöber method, and the following surface modification process involved the use of a silver nitrate aqueous solution. The morphology of BGNs before and after surface modification was observed using field emission scanning electron microscopy (FE-SEM), and the composition of the BGNs was analyzed with energy-dispersive X-ray spectroscopy (EDS). The possible presence of crystalline phases was investigated by X-ray diffraction (XRD) and Fourier-transform infrared spectroscopy (FTIR). Moreover, the *in vitro* mineralization and the antibacterial activity of Ag-BGNs were evaluated.

## 2. Results and Discussion

**Figure 1:**
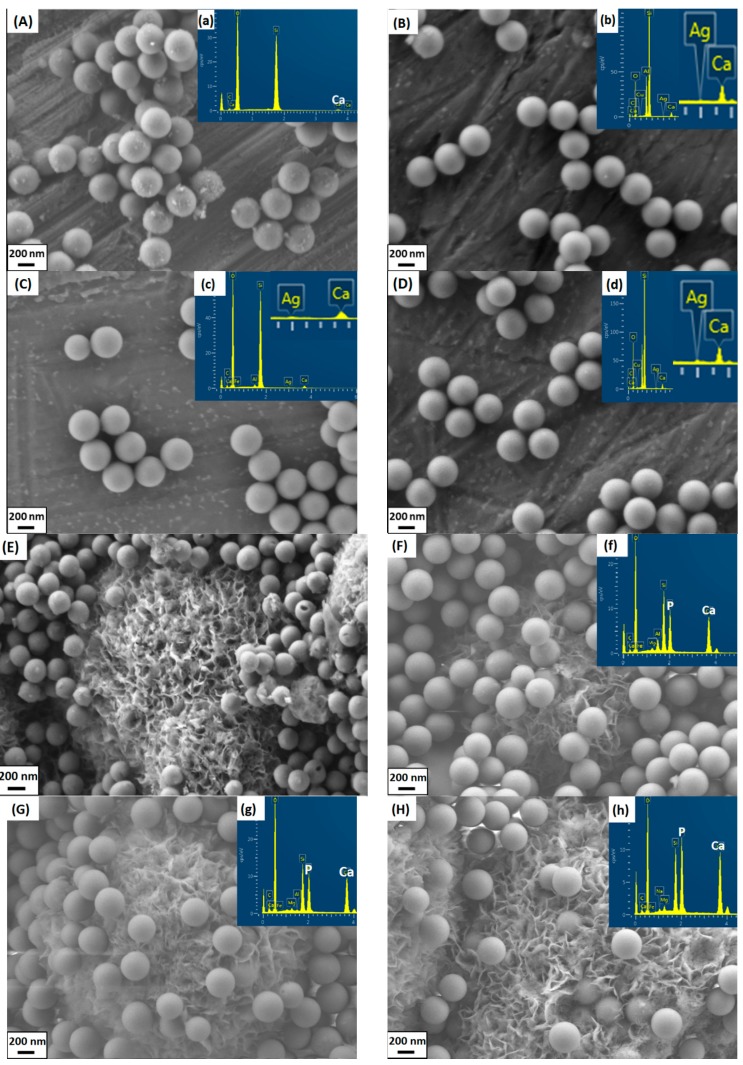
Scanning electron microscopy (SEM) images of samples before and after soaking in SBF for 7 days. (**A**) BGNs; (**B**) Ag-25; (**C**) Ag-60; (**D**) Ag-80; (**E**) BGNs 7d; (**F**) Ag-25 7d; (**G**) Ag-60 7d; (**H**) Ag-80 7d. Inserted are the corresponding energy-dispersive X-ray spectroscopy (EDS) results.

The as-synthesized BGNs were monodispersed and showed a spherical shape (Figure 1a), which is the typical morphology of particles synthesized by the Stöber method [6]. After surface modification with Ag nitrates, all the particles still maintained the spherical shape (Figure 1). Additionally, the sizes of BGNs and all Ag-BGNs were comparable (Table 1). The particles modified at different temperatures showed a similar spherical shape, surface morphology and particle size, indicating that the surface modification did not affect the key characteristics of particles.

Table 1 lists the compositions and particle size of the obtained BGNs. As can be seen, Ag was incorporated in BGNs successfully. Different possible mechanisms can be considered to describe the incorporation of Ag in BGNs. Firstly, positively charged Ag^+^ ions can be adsorbed on negatively charged BGNs through electrostatic interactions. In addition, Ag^+^ ions from the aqueous solution could be incorporated by an ion exchange process with calcium ions in BGNs [18,19]. Moreover, Ag^+^ ions may diffuse into the BGNs, as particles synthesized by the Stöber method contain nanopores on the surface [20]. Notably, the content of Ag in BGNs increased with increasing temperature. This phenomenon is likely due to the higher diffusion mobility of Ag^+^ ions at higher temperatures. It is important to notice that the final composition of BGNs was different from the nominal composition. This result could be attributed to the high solubility of calcium nitrate during the period of reaction and the further loss of calcium caused by washing, which is consistent with the results from previous studies [6,21].

**Figure 2:**
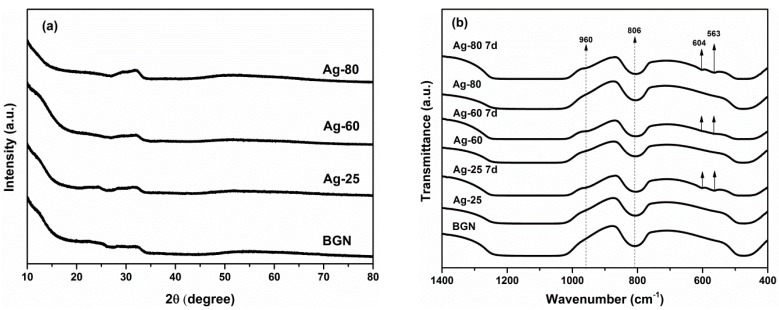
(**a**) X-ray diffraction (XRD) patterns of BGNs and surface modified Ag-BGNs; (**b**) Fourier-transform infrared spectroscopy (FTIR) spectra of Ag-BGNs before and after soak in SBF for 7 days.

A broad shoulder starting at 2θ = ~20° can be seen in the XRD patterns (Figure 2a) of all samples, which is a typical characteristic peak for amorphous silicate materials [6]. Another broad band can be seen at around 32°, which could be attributed to amorphous calcium silicate species [22]. No significant differences could be found in the XRD patterns of Ag-BGNs. These results suggest that particles did not change their amorphous feature, and no silver-related crystalline phase was developed after surface modification. As can be seen in the FTIR spectra (Figure 2b), BGNs and Ag-BGNs show the characteristic bands of silicate glasses. A wide band located in the range 1000–1200 cm^−1^ could be attributed to Si–O–Si symmetric stretching vibration. Bands at 806 and 474 cm^−1^ are ascribed to symmetric stretching and rocking mode of Si–O–Si, respectively [15,23,24]. All the above results indicate that the surface modification did not influence the structure; thus, it was expected that Ag-BGNs could maintain the properties of BGNs, e.g., bioactivity.

Crystals exhibiting the typical morphology of HA [24] could be seen both on BGNs and Ag-BGNs after soaking in SBF for 7 days (Figure 1E–H). It should be noted that some BGNs were located inside the crystals while some BGNs were present on the top of the crystals. During immersion in SBF, HA crystals firstly formed around BGNs, and then the crystals continued forming and growing all throughout the immersion period. However, considering the small size of the BGNs, HA crystals did not form a typical cauliflower-like morphology on the surface of BGNs. The large apatite crystals could only cluster themselves and grow beyond nanoparticles. In this study, BGNs inside the crystals should therefore induce HA formation, while the particles on the top of the apatite crystals are believed to be caused by the processes of sample preparation for SEM (after washing and centrifugation, some individual BGNs without well-exposure to SBF were detached from BGN-apatite clusters and were then placed on top of the crystals). Similar SEM images of BGNs after soaking in SBF can be found in previous reports [25,26]. In addition, P could be detected on Ag-BGNs after soaking in SBF (Figure 1). The Ca/P ratio of the crystals formed on Ag-BGNs was near 1.64, which is close to the Ca/P ratio of stoichiometric HA. In addition, two new bands at around 563 and 604 cm^−1^ can be seen in the FTIR spectra (Figure 2b) of Ag-BGNs soaked in SBF for 7 days. These two bands are characteristic bands of HA, which could be attributed to P–O asymmetric bending in apatite crystals [24]. Based on the SEM, FTIR, and EDS results, it can be concluded that Ag-BGNs were bioactive in the sense that HA crystals could form on them upon soaking in SBF for 7 days.

**Figure 3:**
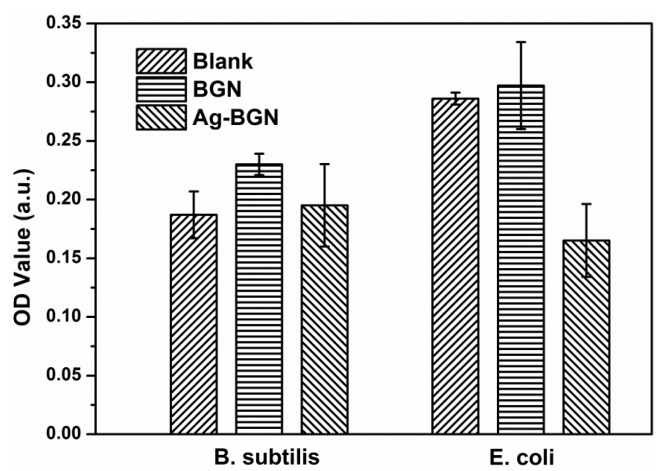
Antibacterial activity of BGNs and Ag-BGNs (Ag-25) towards *B. subtilis* and *E. coli*.

Ag-25 was selected for antibacterial tests as it had the lowest content of Ag (Table 1). Higher Ag contents should lead to stronger antibacterial activity, but they present possible cytotoxic effects. The results of antibacterial tests are shown in Figure 3. As expected, BGNs did not show antibacterial effects on both *E. coli* and *B. subtilis* As a comparison, Ag-25 exhibited antibacterial effects on *E. coli*, while no effects towards *B. subtilis* could be observed. This result could be due to the low concentration of Ag in Ag-25, and the released Ag^+^ ions could not reach the minimum inhibitory concentration of *B. subtilis*. Previous research has shown that Gram-negative *E. coli* was more susceptible to silver than Gram-positive Staphylococcus aureus due to the difference in cell walls structure [27,28]. Considering the antibacterial effects of Ag-25, it can be concluded that all modified Ag-BGNs produced in this study should possess antibacterial capability.

As silver nanoparticles could induce toxicity towards cells [29], Ag-doped BGs could also cause cytotoxic effects. It has been reported that the cytotoxicity of Ag-doped BGs depended on the used concentration of materials and on the culture time [30]. To the best of our knowledge, comprehensive reports on the cytotoxicity of Ag-containing BGNs have not been published so far. The cytotoxicity of Ag-BGNs should also depend on the used concentration and culture time. The detailed biocompatibility assessment and the biological properties of Ag-BGNs will be investigated in further studies.

## 3. Materials and Methods

### 3.1. Synthesis of BGNs 

BGNs were synthesized by a modified Stöber method. Briefly, one solution containing 25 mL ethanol (VWR, Darmstadt, Germany) and 3 mL tetraethyl orthosilicate (Sigma-Aldrich, Munich, Germany) was added into another solution containing distilled water (12.4 mL), ethanol (8.12 mL), and ammonia (4.5 mL, 28%, VWR) under continuous stirring. After 30 min of reaction, 1.35 g calcium nitrate tetrahydrate (CaNO_3_·4H_2_O; Sigma-Aldrich) was added into the suspension and stirred for further 1.5 h before being centrifuged and washed twice with water and once with ethanol. The collected deposits were dried at 60 °C for 4 h, followed by calcination at 700 °C for 2 h with a heating rate of 2 °C/min.

### 3.2. Surface Modification

The as-prepared BGNs (0.5 g) were soaked in 20 mL of a 0.5-M silver nitrate (AgNO_3_; Sigma-Aldrich) aqueous solution at 25, 60, and 80 °C for 6 h, respectively. The suspension was then centrifuged and washed twice with distilled water. The obtained wet powders were dried at 60 °C for 3 h and then sintered at 560 °C for 1 h with a rate of 2 °C/min to stabilize the structure and to remove residual nitrates. The obtained samples were named Ag-25, Ag-60, and Ag-80 according to the temperatures used (25, 60, and 80 °C).

### 3.3. Characterization

The morphology of Ag-BGNs was observed by FE-SEM (Auriga, Carl-Zeiss, Jena, Germany). The particle size was determined from SEM images, measuring around 100 particles by means of ImageJ software (National Institutes of Health, Bethesda, MD, USA). The composition of particles was investigated by EDS. FTIR (Nicolet 6700, Thermo Scientific, Schwerte, Germany) was performed using the KBr (Merck, Darmstadt, Germany) pellet method. The weight ratio of sample to KBr was set at 1:100. For the analysis, 32 scans at a resolution of 4 cm^−1^ were performed in the wavenumber range 2000–400 cm^−1^. A Bruker D8 Discover diffractometer (Bruker, Karlsruhe, Germany) with Cu Kα radiation at 40 kV and 30 mA was used to record XRD patterns in a 2θ range of 10°–80° with a step size of 0.014°/step.

### 3.4. In Vitro Mineralization

The *in vitro* mineralization of Ag-BGNs was investigated by soaking samples in SBF [31] at a ratio of 1 mg/mL for 7 days. The samples were kept in an incubator at 37 °C and 90 rpm for up to 7 days. At pre-determined time points, the suspension was centrifuged, washed twice with distilled water, and then dried at 60 °C for 3 h. Apatite formation on the samples was evaluated using FTIR, FE-SEM, and EDS.

### 3.5. Antibacterial Assessment

Ag-25 was selected for antibacterial tests given its lowest amount of Ag. The test was carried out as described in the literature [32]. Briefly, Ag-25 was immersed in phosphate buffered saline for 24 h at a concentration of 1 mg/mL at room temperature. PBS was used as a blank control and BGNs as a negative control. Before assessment, Gram-positive *B. subtilis* and Gram-negative *E. coli* were cultured in the nutrient for 24 h at 37 °C. Wells of a *sterilized* 96-well plate were inoculated with 50 μL of the particle suspension and 50 μL of bacterial suspension for 16 h. Tests were conducted in quadruplicate. The optical density of 600 nm (OD 600) was measured spectrophotometrically with a microplate reader (Biotek, Bad Friedrichshall, Germany). Since the optical density might have interfered with the samples, the OD value of the samples before culture was removed from the final results as the background.

## 4. Conclusions

Monodispersed, bioactive Ag-BGNs were successfully synthesized by a modified Stöber method followed by surface modification at relatively low temperatures (25 to 80 °C) to incorporate Ag. The particle size was around 370 nm. With the rise of temperature, the incorporated Ag content increased. The modification had no obvious influence on the morphology, size, and structure of particles. HA formed on Ag-BGNs upon soaking in SBF for 7 days. Antibacterial tests showed that the presence of Ag-BGNs (Ag-25) could inhibit the growth of *E. coli*. The results have demonstrated a facile surface modification method to incorporate Ag into bioactive glass nanoparticles. The modified BGNs remain monodisperse, are bioactive, and acquire antibacterial capability. Ag-BGNs are thus promising materials for biomedical applications.

## Figures and Tables

**Table 1:** Composition determined by energy-dispersive X-ray spectroscopy (EDS) and particle size of non-modified and Ag-modified bioactive glass nanoparticles (BGNs).

Name	Composition (mol%)	Particles Size (nm)
BGN	96.60SiO_2_-3.40CaO	370 ± 35
Ag-25	95.61SiO_2_-4.26CaO-0.12Ag_2_O	365 ± 23
Ag-60	95.46SiO_2_-4.35CaO-0.19Ag_2_O	373 ± 25
Ag-80	95.69SiO_2_-4.07CaO-0.24Ag_2_O	367 ± 24
